# Protein biomarkers in serum as a conservation tool to assess reproduction: a case study on brown bears (*Ursus arctos*)

**DOI:** 10.1093/conphys/coab091

**Published:** 2021-12-06

**Authors:** Abbey E Wilson, Sarah A Michaud, Angela M Jackson, Gordon Stenhouse, Cameron J R McClelland, Nicholas C Coops, David M Janz

**Affiliations:** 1 Department of Veterinary Biomedical Sciences, University of Saskatchewan, 52 Campus Drive, Saskatoon, Saskatchewan S7N 5B4, Canada; 2 The University of Victoria Genome BC Proteomics Centre, 4464 Markham St #3101, Victoria, British Columbia V8Z 7X8, Canada; 3 Grizzly Bear Program, fRI Research, 1176 Switzer Drive, Hinton, Alberta T7V 1V3, Canada; 4 Department of Forest Resource Management, University of British Columbia, 2424 Main Mall, Vancouver, British Columbia V6T 1Z4, Canada

**Keywords:** wildlife management, reproduction, proteomics, population monitoring, physiology, conservation

## Abstract

Monitoring the reproductive characteristics of a species can complement existing conservation strategies by understanding the mechanisms underlying demography. However, methodology to determine important aspects of female reproductive biology is often absent in monitoring programs for large mammals. Protein biomarkers may be a useful tool to detect physiological changes that are indicative of reproductive state. This study aimed to identify protein biomarkers of reproductive status in serum collected from free-ranging female brown bears (*Ursus arctos*) in Alberta, Canada, from 2001 to 2018. We hypothesized that the expression of proteins related to reproduction in addition to energetics and stress can be used to answer specific management-focused questions: (i) identify when a female is pregnant, (ii) detect if a female is lactating, (iii) determine age of sexual maturity (i.e. primiparity) and (iv) assess female fertility (i.e. reproduction rate). Furthermore, we investigated if silver spoon effects (favourable early life conditions provide fitness benefits through adulthood) could be determined using protein expression. A target panel of 19 proteins with established relationships to physiological function was measured by peptide-based analysis using liquid chromatography and multiple reaction monitoring mass spectrometry and their differential expression was evaluated using a Wilcoxon signed-rank test. We found biomarkers of pregnancy (apolipoprotein B-100 and afamin), lactation (apolipoprotein B-100 and alpha-2-macroglobulin) and sexual maturity (corticosteroid-binding globulin), but there were no statistically significant relationships with protein expression and fertility. The expression of proteins related to reproduction (afamin) and energetics (vitamin-D binding protein) was associated with the nutritional quality of the individual’s present habitat rather than their early life habitat. This study highlights potential biomarkers of reproductive status and provides additional methods for monitoring physiological function in wildlife to inform conservation.

## Introduction

Large carnivores are a key component of biodiversity that are facing increasing pressure from the cumulative impacts of a growing human population (Ripple *et al.*, 2014). They play critical roles in the functioning of natural ecosystems, generate economic revenues and are of great cultural value at local and global scales, making them a high priority for conservation (Bruskotter *et al.*, 2017). However, many populations continue to decline resulting in the need for additional management and monitoring approaches (Dickman *et al.*, 2011). Conservation and management strategies of brown bear (*Ursus arctos*) populations have evolved to include a variety of population assessment tools including using barbed wire hair snags to determine abundance and distribution of individuals (Boulanger *et al.*, 2018), fitting animals with satellite GPS-collars that provide detailed spatial data for each individual to identify habitat use (McClelland *et al.*, 2020) and developing complex models to predict survival and habitat selection (Nielsen *et al.*, 2010). To complement these features of brown bear populations (e.g. abundance and density, demographic proportions and rates), a better understanding of the health and reproductive performance of individuals will aid in determining key attributes of the performance of populations and their ability to persist (Wilson *et al.*, 2020a). With some populations residing at critically low densities, such as the Apennine brown bear (*Ursus arctos marsicanus*) population in central Italy (Ciucci *et al.,* 2015) and the Gobi bear (*Ursus arctos gobiensis*) population in Mongolia (Tumendemberel *et al.,* 2019), improving the effectiveness of large carnivore conservation and traditional monitoring methods depends on developing tools that will help managers understand the performance of populations.

One measure of population performance is the estimation of reproductive rates at the population level. This can be measured by parameters such as demographics, productivity and reproductive characteristics, which includes sperm production, age of sexual maturity and body size in males and age of primiparity, litter size, interval between births, reproductive rate and cub survival rate in females (Garshelis *et al.,* 2005). General female reproductive characteristics have been described in detail for Western Europe and North America populations of brown bears (Steyaert *et al.,* 2012). The mating season for brown bears in our study area extends from mid-May to the end of July and peaks in mid-June (Stenhouse *et al.,* 2005). After successfully mating, females enter a phase of embryonic diapause (delayed implantation), in which the fertilized ova remain dormant in the uterus for 5–6 months prior to implantation in November or December (Tsubota and Kanagawa, 1993; Mano and Tsubota, 2002; Friebe *et al.,* 2014) at which time bears typically enter the den, making it difficult to detect pregnancy. Female bears give birth to cubs in the den after a gestation period of 6–8 weeks between January and March and will lactate for the following 1.5–2.5 years (Farley and Robbins, 1995; Oftedal, 2000; Steyaert *et al.,* 2012). However, bears and other mammalian species experience pseudopregnancy, where non-pregnant individuals exhibit nesting, weight gain, mammary enlargement, lactation and maternal behaviour (Gobello *et al.,* 2001; Schulz *et al.,* 2003; Steyaert *et al.* 2012), further limiting our ability to determine pregnancy and lactation status. There is also uncertainty in many aspects of reproductive biology in female brown bears, such as the age of sexual maturity, which although commonly assumed to be ~5 years old (Støen *et al.,* 2006; Mace *et al.* 2012), may be 4 years (Stenhouse, unpublished data) or as young as 3 years (Frković *et al.,* 2001; Zedrosser *et al.,* 2004) in some populations. Furthermore, reproductive rates and biological fitness of individual animals can be impacted by compromised health (Eberhardt, 2002; Zedrosser *et al.,* 2013), which can in turn be influenced by a number of environmental conditions including those related to food availability and anthropogenic disturbance (Wilson *et al.,* 2021). Early life environments can influence how individuals develop (e.g. growth, fat deposition and organ development) and have fitness implications that can ultimately affect population growth rates (Lindström, 1999; Pigeon *et al.,* 2019). The silver spoon hypothesis states that individuals developing under favourable conditions (i.e. higher food resources) will have greater fitness benefits (Wilkin and Sheldon, 2009), which can influence reproductive rates. Although this information is critical to population management, these parameters can be difficult to measure due to the complex reproductive characteristics of this species.

Methods for determining reproductive status and performance can be time intensive and costly. Such techniques include immobilizing and radio-collaring females to observe their fate and reproductive performance (Zedrosser *et al.,* 2009; Mace *et al.,* 2012), counting cubs from an aircraft to examine cub survivorship and litter size (Miller *et al.,* 2003) and extracting DNA from hair samples to determine identity and genetic relationships (Zedrosser *et al.,* 2004; Proctor *et al.,* 2012; Phoebus *et al.,* 2020). Identifying physiological biomarkers in biological samples (i.e. blood, hair and skin) that can help to identify and forecast reproductive status, the ability of individuals to reproduce and the number of offspring that may be produced may address the need for novel approaches to monitoring the productivity and reproductive characteristics of populations. Advanced proteomic tools have recently been used to understand the mechanisms related to reproduction (e.g. gametogenesis and fertilization) and identify potential biomarkers of infertility in humans (Panner Selvam *et al.,* 2019). However, only a few studies have applied similar techniques to assess reproductive status and function in wildlife, including those to identify protein biomarkers indicative of pregnancy in cheetahs (Koester *et al.,* 2017), several wild canid species (Bauman *et al.,* 2008), black bears (Foresman and Daniel Jr, 1983) and polar bears (Curry *et al.,* 2012). While specific proteins related to reproduction may provide insight on reproductive status, changes in key physiological mechanisms related to metabolism and stress can also influence reproductive performance (Edwards *et al.,* 2019). Proteins are functional molecules that respond to stimuli and help to regulate biological function via information expressed in the genome and manifested at the message level (mRNA; Cox and Mann, 2007). More specifically, proteins in serum have been secreted or leaked from both tissue and blood cells (Hu *et al.*, 2006), which makes the serum proteome dynamic and complex, but also facilitates the detection of protein biomarkers that represent different physiological and pathological conditions (Ray *et al.,* 2011). Blood collection is not always feasible or preferred in free-ranging animals due to the negative impacts of capture and handling on individual health (Cattet *et al.,* 2008). However, initially identifying biomarkers in serum samples collected from individuals with known reproductive history will facilitate the detection of proteins related to reproduction that may be detectable in other sample matrices that can be collected non-invasively (e.g. hair and skin) in the future (Wilson *et al.* 2020a, 2020b).

This study aimed to identify potential biomarkers of reproduction in serum collected from individual brown bears. We utilized a long-term database of serum samples collected from free-ranging female brown bears residing in Alberta, Canada, and systematically chose samples that would be useful in answering specific management-focused research questions. We hypothesized that the expression of proteins related to energetics, reproduction and stress can be used to assess specific aspects of reproductive function including pregnancy, sexual maturity and reproduction rate (i.e. fertility). Our specific research objectives were to investigate if we can use biomarkers in serum to (i) identify when a female is pregnant, (ii) detect if a female is lactating, (iii) determine age of sexual maturity (when reproduction is possible), (iv) assess female fertility (high or low reproducers) and (v) test the silver spoon hypothesis (daughters that develop under favourable landscape conditions will have greater expression of proteins indicative of higher reproductive performance; Wilkin and Sheldon, 2009). Given that specific proteins related to reproduction in our target panel have been used as biomarkers of pregnancy and fertility in other species (Curry *et al.,* 2012; Dieplinger and Dieplinger, 2015; Dietzel *et al.,* 2016; Floeh *et al.,* 2016; Linder, 2016; Ricklin *et al.,* 2016), we predicted that the expression of these proteins would be greater in pregnant individuals as well as individuals that produced several cubs over the monitoring period (high reproducers). Lactation and sexual maturity require changes in metabolic (Chinn *et al.,* 2018) and reproductive condition (Dettmer *et al.,* 2015), respectively, which we predict would be associated with fluctuations in biomarkers related to energetics, reproduction and/or stress. We predicted that cubs residing in home ranges with high nutritional quality (greater density kilocalories/km^2^) during early life (based on an assessment of the mother’s home range) would have a higher concentration of proteins related to reproduction and energetics combined with lower expression of stress proteins, as greater food availability has been shown to facilitate reproduction and decrease stress (Bryan *et al.,* 2013; Ferkin, 2017; Wilson *et al.,* 2020a). Using protein expression as a measure of fitness may provide a new methodology for determining silver spoon effects by using samples collected non-invasively in the future (e.g. hair and skin), rather than approaches that require the capture and handling of individuals to determine traditional measurements of fitness, such as body condition and mass (Nielsen *et al.,* 2013).

## Materials and methods

### Study animals

The free-ranging brown bear population in Alberta, Canada, has been monitored by the fRI Research Grizzly Bear Program over the past 23 years, resulting in an extensive database of individual identities with detailed spatial data, genetic relationships and observed cubs on the landscape. This population resides in parks and protected areas in the mountainous (up to 3500 m elevation) region to the west as well as lower elevation areas in the foothills to the east, which are subject to anthropogenic resource use, including forestry, oil and gas exploration and mining (Berland *et al.,* 2008; Proctor *et al.,* 2012). The forest conditions and food resources for this brown bear population have been documented extensively (Nielsen *et al.*, 2004b; Stenhouse *et al.,* 2005; Nielsen *et al.,* 2010; Bourbonnais *et al.,* 2013). Initial population inventory studies on these populations revealed fewer brown bears than expected (Boulanger *et al.,* 2005b) and, along with other factors, resulted in the province classifying this species as threatened in 2010 (Festa-Bianchet, 2010; Clark and Slocombe, 2011). However, inventory work completed in the same areas 10–13 years after the initial count demonstrated that the population doubled in number over this period of time (Stenhouse *et al.,* 2015, 2020, 2021), suggesting an increase in survival and/or reproductive performance. In addition to population surveys, the long-term monitoring of this population also included recording productivity, survival and health, determining movement and behaviours and identifying habitat use and selection.

### Sample collection

We used serum samples collected from the free-ranging brown bear population in Alberta, Canada. These samples had been collected by fRI Research during routine capture operations as part of their long-term monitoring program. Details regarding the handling and sampling of bears can be found in Cattet *et al.* (2008). Blood was collected from the femoral vein into sterile tubes and centrifuged within 8 hours of collection to extract the serum. Samples were immediately frozen at −20°C until they were ultimately transferred to −80°C for long-term storage. All captures were authorized by Alberta Environment and Parks and Parks Canada and research and collection permits were obtained on an annual basis. All capture and handling procedures were based on the Canadian Council on Animal Care and the American Society of Mammologists guidelines (Rowsell, 1991; Sikes, 2016) and were approved annually by the University of Saskatchewan’s Committee on Animal Care and Supply and by the Alberta Environment and Parks Animal Care Committee (Animal Use Protocol Number 20010016).

### Sample selection

Samples were systematically chosen from the long-term database at fRI Research in accordance with our directed research questions for determining reproductive biomarkers in serum of brown bears. A total of 77 samples collected from 48 individuals residing in five bear management areas (BMAs; Castle, Clearwater, Grand Cache, Swan Hills, Yellowhead) in Alberta during 2001–2018 were chosen for this study. Samples were collected during the active season (April–October), with the majority of samples collected in the spring (April–14 June; *n* = 47) and fewer in the summer (15 June–07 August; *n* = 9) and fall (08 August–October; *n* = 21), as defined by brown bear feeding habits in our study area (Nielsen *et al.,* 2004a). In order to answer these questions as efficiently as possible, the same sample may have been used to address multiple objectives. For example, one sample from an adult, non-pregnant female was used as an adult for the sexual maturity objective as well as a control for the pregnancy status objective. The description and number of samples for each objective are summarized in Table 1.

**Table 1:** Selection of serum samples collected from free-ranging female brown bears in Alberta, Canada, to determine if expression of proteins can be used to assess reproductive function

Objective	Sample description	Number of samples
Pregnancy	Identified years that a female was assumed to be pregnant, based on observation date of cub of the year, yearling or two-year-old with female	Pregnant: 20 (17 individuals) Non-pregnant: 20
Lactation	Visual observation (or not) of lactation during capture of adult females	Lactation: 22 (19 individuals) Non-lactation: 14
Sexual maturity	Determined age and age class (yearling, 1 year old; sub-adult, ≤4 years old; adult, ≥5 years old) from birth year provided in database^a^; chose two samples from each individual that spanned the years from sub-adult to adult	Sub-adult to adult: 20 (10 individuals)
Fertility	Determined number of cubs for each adult female from visual observation (capture, helicopter, ground, photo), genetics, GIS clusters and/or denning behaviour per year monitored and classified females as high reproducers (>0.73 cubs) or low reproducers (<0.73 cubs; samples from bears that had zero cubs were included if no cubs were visually observed)	35 (35 individuals) High reproducers: 14 Low reproducers: 21
Silver spoon	Determined daughters for each individual from our DNA database^b^; chose one sample from the most recent year for each mother/daughter pair	25 (25 individuals) Mother/daughter pairs: 11

a ^a^The ages of all bears were estimated by counting the cementum annuli of an extracted premolar extracted during capture operations (Stoneberg and Jonkel, 1966; Matson *et al.,* 1993; Cattet *et al.,* 2018).

b ^b^Our DNA database is comprised of samples from population inventory efforts across the bear management areas in Alberta over several years (Boulanger *et al.,* 2005a, 2005b, 2018; Stenhouse *et al.,* 2015).

### Pregnancy and lactation

To identify if a female was pregnant, we identified years that a female was assumed to be pregnant, based on observation date of cub of the year, yearling or 2-year-old and a sample was included if the sample year and assumed pregnant year matched. Samples from assumed to be pregnant females were separated by spring and summer (*n* = 23 in April–July) and fall (*n* = 17 in August–October), as these two seasonal time points likely reflect different stages of pregnancy (before and after implantation). The ages of pregnant individuals ranged from 3 to 4 years (*n* = 3), 5 to 7 years (*n* = 9) and 8 to 20 years (*n* = 8). Non-pregnant samples were chosen from adult females (*n* = 20). A female was assumed to be not pregnant if she was observed lactating at the time of sampling (*n* = 3), with cubs at the time of sampling (*n* = 12) or if she was observed with no cubs 1 year after sampling (*n* = 5). To detect if a female is lactating, we identified visual observation of lactation status during capture and chose samples with a matching sample year and known lactation status. Adult females that were not observed lactating were classified as non-lactating. Bears can experience a pseudopregnancy and may lactate due to physiological changes similar to pregnancy (Creel *et al.,* 1991; Gobello *et al.,* 2001; Schulz *et al.,* 2003), thus we could not determine if the female was lactating as a result of producing cubs or as a result of being pseudopregnant (considered to be non-pregnant because cubs are not produced). A high prevalence of physiological and behavioural changes due to pseudopregnancy has been reported in domestic dogs (Root *et al.,* 2018) and several studies have been conducted to differentiate between pregnant and pseudopregnant bears in captivity, as this phenomenon occurs fairly frequently in bears (Durrant *et al.,* 2006; Stoops *et al.,* 2012; Bryant and Roth, 2018). However, the frequency at which free-ranging brown bears become pseudopregnant is unknown.

**Table 2:** Target proteins identified and quantified in serum collected from free-ranging female grizzly bears in Alberta, Canada, from 2001–2018

Category	Protein	Biomarker
Energetics	Adiponectin	Metabolic disease, protein-calorie malnutrition and liver disease
	Clusterin	
	Apolipoprotein B-100	
	Alpha-1-acid glycoprotein	
	Transthyretin	
	Vitamin D-binding protein	
Reproduction	Ceruloplasmin	Pregnancy and fertility
	Fetuin-B	
	Complement C3	
	Afamin	
Stress	Corticosteroid-binding globulin	Acute and chronic inflammation
	Alpha-2-macroglobulin	
	Kininogen-1	

See Wilson *et al.* (2020b) for more details and references for the specific biological functions of each protein.

### Sexual maturity

To determine age of sexual maturity (when reproduction is possible), we first determined age and age class (yearling, 1 year old; sub-adult, ≤4 years old; adult, ≥5 years old) from the birth year provided in our database. The ages of all bears were estimated by counting the cementum annuli of an extracted premolar extracted during capture operations (Stoneberg and Jonkel, 1966; Matson *et al.,* 1993; Cattet *et al.,* 2018). Next, we determined age range relative to years that samples were collected and chose two samples from each individual that spanned the years from sub-adult to adult. Individuals included in this sample set were sampled at ages 4 and 5 (*n* = 5), 4 and 8 (*n* = 1), 3 and 7 (*n* = 1), 4 and 6 (*n* = 1), 4 and 7 (*n* = 1) and 3 and 5 (*n* = 1) years.

### Fertility

To assess female fertility (i.e. whether females produced a high or low number of cubs), we first determined the number of cubs for each individual from our database using visual observation (capture, helicopter, ground, photo), genetics, geographic information system (GIS) clusters and/or denning behaviour and divided this number by the number of years the individual was monitored to provide a numerical value for the number of cubs per year monitored. The mean number of cubs produced per year monitored was 0.73 (range: 0–2 cubs per year monitored); therefore, high reproducers were defined as females that had >0.73 cubs per year monitored (*n* = 14; range: 0.83–2.00 cubs per year monitored), while low reproducers were defined as females that had <0.73 cubs per year monitored (*n* = 21; range: 0.00–0.67 cubs per year monitored; samples from bears that had zero cubs were included if no cubs were visually observed). All samples were chosen from adult females that were not assumed to be pregnant.

### Silver spoon effects

To test the silver spoon hypothesis (daughters that develop under favourable landscape conditions, similar to their mothers, will have greater expression of proteins related to reproduction), we first identified daughters for each individual from our DNA database. Our DNA database is comprised of samples from population inventory efforts across the bear management areas in Alberta over several years (Boulanger *et al.,* 2005a, 2005b, 2018; Stenhouse *et al.,* 2015). We chose a sample from the most recent year for each mother/daughter pair and extracted the kilocalorie (hereafter Kcal) density as a proxy for nutritional quality for the home ranges of each mother/cub pair (see kilocalorie per home range for silver spoon analysis section below). All cubs were classified as adults (≥5 years old), except for two cubs that were estimated to be 3 and 4 years old at the time of sampling. When applicable, we chose the most recent sample from each individual for analysis to ensure the highest sample quality possible, as samples were collected and stored over several years.

### Liquid chromatography and multiple reaction monitoring mass spectrometry assay for targeted analysis

The current study utilized a proteomic profile developed from our previous work (Wilson *et al.,* 2020b) that targeted proteins related to energetics, reproduction and stress in the skin of brown bears residing in Alberta, Canada. All protein extraction and digestion methods as well as the liquid chromatography and multiple reaction monitoring mass spectrometry (LC-MRM/MS) methods were completed at The University of Victoria Genome BC Proteomics Centre (Victoria, British Columbia, Canada) following previously described protocols (see Wilson *et al.,* 2020b). Proteins were digested using trypsin (5 μl at 1 mg/ml; Worthington Biochemical Corporation, Lakewood, NJ, USA) at a 20:1 substrate:enzyme ratio. A mix of light peptides was used to prepare the standard curve and stable isotope-labelled standard (SIS) peptides were used as internal standards for both the standard curve and samples.

A panel of 19 proteins with established relationships to physiological function was quantitated by peptide-based analysis using LC-MRM/MS (Table 2). A total of 54 peptides representing 19 target proteins were selected, and each peptide assay was validated in a representative serum sample. Details regarding the methodology for development and validation of the peptide panel as well as the LC-MRM/MS targeted assay parameters are provided elsewhere (Wilson *et al.,* 2020b). In short, tryptic digests were analysed using an LC coupled to a triple quadrupole mass spectrometer (Agilent 1290 and 6495, respectively; Agilent Technologies, Santa Clara, CA, USA). Peptides were empirically optimized by analysis of the purified SIS peptides using Skyline-daily Quantitative Analysis software (version 19.1.1.248, MacCoss Laboratory, University of Washington, Seattle, WA, USA). The standard curve was used to calculate the peptide concentration in fmol/μl of serum in samples through linear regression using the endogenous to SIS peak area ratios. All peptide sequences were identified from the *Ursus* family (*Ursus maritimus* and *Ailuropoda melanoleuca*) in the UniProt database (Hinxton Cambridge, UK; Bateman, 2019), as the brown bear proteome is not fully sequenced and therefore not fully represented in Uniprot. A total of 22 (out of 54) peptides were removed from analysis because >25% of values were either below the assay’s lower limit of quantitation (LLOQ) or above the assay’s upper limit of quantitation (ULOQ), resulting in 6 proteins removed from the analysis (78 kDa glucose-regulated protein, annexin, endoplasmin, prostaglandin F synthase 1, serpin B5 and superoxide dismutase). The remaining 13 proteins had at least one proteotypic peptide with all sample values falling between the LLOQ and the ULOQ and where multiple peptides were available for a single protein, the protein was represented by the peptide with the greatest average concentration across all samples.

### Kilocalorie per home range for silver spoon analysis

We created a single Kcal layer by combining total Kcals of ungulates (moose, sheep and elk), fruit species, herbaceous species, root species and ants. Individual food Kcal layers were made available through our previous work (McClelland *et al.,* 2021) and were created using species biomass layers converted to Kcal content using Kcal values per food type (López-Alfaro *et al.,* 2015; Nielsen *et al.,* 2017). We calculated annual brown bear home ranges using minimum convex polygons where only 95% of locations were used, to account for outliers. Home ranges were created using the ‘adehabitatHR’ package (Calenge, 2006) in R statistical software and bears with <4 months of data within a single year were omitted from this analysis (*n* = 3 mother/daughter pairs; *n* = 7 samples). We then extracted the total Kcal content per home range, and to allow comparability between home ranges, calculated Kcals per km^2^ by dividing total Kcals by home range area. Since GPS collars were fitted at the time of sampling, it was assumed that the landscape data collected throughout the home range of an individual following the sampling event was similar to the home range when the sample was collected (Smulders *et al.,* 2012; Sorensen *et al.,* 2015).

### Statistical analysis

Physiological biomarkers are commonly identified by comparing the mean and variance of protein expression between distinct treatment groups (e.g. diseased vs. healthy, pregnant vs. non-pregnant) using a paired or unpaired *t*-test and/or analysis of variance (Dai and Lu, 2012). Given the limited sample size in the current study and non-normal distribution of protein expression, a Wilcoxon signed-rank test was used to compare the expression of the 13 target proteins between the two groups within each objective (pregnancy, lactation, sexual maturity, fertility and silver spoon). We specifically tested whether the median of the positive group (e.g. pregnant) was greater than the median of the negative group (e.g. non-pregnant) for all objectives. We used a Pearson correlation to determine if the expression of proteins were correlated with the specific numerical data of each objective (e.g. age, Kcal density and number of cubs produced per year monitored). We also used pair-plots (Pearson r ≥ 0.70) to ensure that expression between different proteins was not collinear and thus we were not measuring the same signal. Given that samples in each group within each objective were distributed throughout the active season (i.e. not all samples within one group were collected within one season), samples were not separated by season for the analysis of biomarkers related to lactation, sexual maturity, fertility and silver spoon.

To determine if there was support for silver spoon effects on protein expression in brown bears, we first divided mothers and cubs into two groups that occupied either high- or low-quality habitat by calculating the mean Kcal per km^2^ from all bears (70 000 000 Kcal/km^2^) and designating high-quality habitat above this threshold and low-quality habitat below. Silver spoon effects occur when favourable early life conditions provide fitness benefits through adulthood, even if conditions worsen (Bonifas and Bouchard, 2021). In the current study, early life condition was represented by the nutritional quality (Kcal density) in the home range of the mother, since the cub typically remains with her for 2–3 years. The protein expression in the sample collected from the cub in her present habitat represents the measure of fitness benefits (protein expression). The median expression of proteins in cubs in their birth (early life) habitat and present habitat were compared between high- and low-quality habitat groups using a Wilcoxon signed-rank test and Pearson correlations as described above. Differences were considered significant when α < 0.05; however, marginally significant (α < 0.1) results are also presented due to the limited sample size in this study. Data were analysed using R statistical software version 4.0.3 and R studio version 1.3.1093 (https://www.R-project.org/) (R statistical software 2020).

**Table 3:** Summary of significantly (+, *P* < 0.1; ++, *P* < 0.05) greater serum expression of proteins related to energetics, reproduction and stress in the positive group within each objective

		Pregnancy	Lactation	Sexual maturity	Fertility	Silver spoon effects
Energetics	Adiponectin			+ ↓		
	Alpha-1-acid glycoprotein				+	
	Apolipoprotein B-100	+; ++ (in fall)	++			
	Clusterin					
	Transthyretin					+ ↑ (present habitat)
	Vitamin D-binding protein	+				++ ↑ (present habitat)
Reproduction	Afamin	++				++ ↑ (present habitat)
	Ceruloplasmin					+ ↑ (present habitat)
	Complement C3			+ ↑		
	Fetuin-B				+ ↓	++ ↑ (overall); + ↑ (birth habitat); + ↑ (present habitat)
Stress	Alpha-2-macroglobulin	+	++			
	Corticosteroid-binding globulin			++ ↑		
	Kininogen					++ ↑ (overall); + ↑ (present habitat)

Positive and negative relationships within each objective are represented by an arrow (↑, positive; ↓, negative) for those proteins that were correlated (Pearson r ≥ 0.70) with numerical data. Within silver spoon effects, present habitat refers to the nutritional quality of the bear’s current habitat, birth habitat refers to the nutritional quality of the bear’s habitat during early life (represented by the nutritional quality of the mother’s home range) and overall refers to the combination of mothers andcubs.

## Results

A total of 77 serum samples collected from 48 individuals were used to determine if the expression of proteins related to energetics, reproduction and stress could be used to assess specific aspects of reproductive function including pregnancy, lactation, sexual maturity, fertility and silver spoon effects. All 13 target proteins were used for each analysis, as no proteins were determined to be collinear (Pearson r ≤ 0.7). The expression of proteins related to energetics, reproduction and stress were indicative of changes in the reproductive status of females, including pregnancy, lactation and sexual maturity, but was not appropriate for investigating fertility or silver spoon effects. The expression of at least one protein related to energetics, reproduction and stress were indicative of changes in the reproductive status of females; however, the expression of adiponectin, alpha-1-acid glycoprotein, clusterin, transthyretin, ceruloplasmin and complement C3 was not significantly (*P* > 0.05) different between groups within any objective. A summary of significant and marginally significant (*P* < 0.1) differences between groups within each objective for the serum expression of proteins related to energetics, reproduction and stress is shown in Table 3.

### Pregnancy and lactation

**Figure 1:**
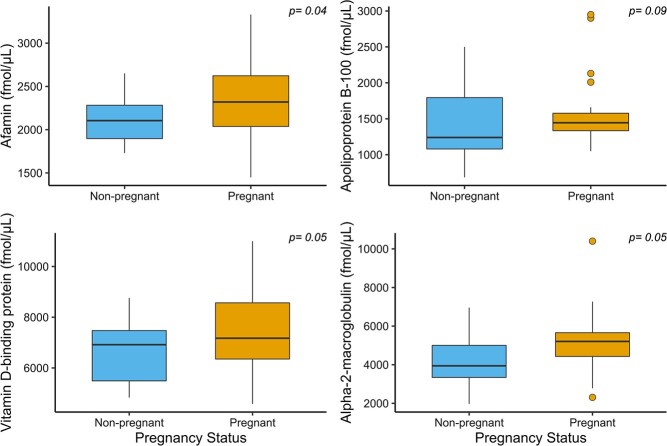
Serum expression of proteins related to energetics, reproduction and stress in female grizzly bears assumed to be non-pregnant (*n* = 20) and pregnant (*n* = 20). The box-and-whisker plots show (i) the median represented by a thick horizontal line; (ii) the interquartile range represented by the box; (iii) the minimum and maximum values, excluding outliers, represented by the lower and upper whiskers; and (iv) outliers being less than or greater than 1.5 times the lower and upper quartiles, represented by the closed circles.

**Figure 2:**
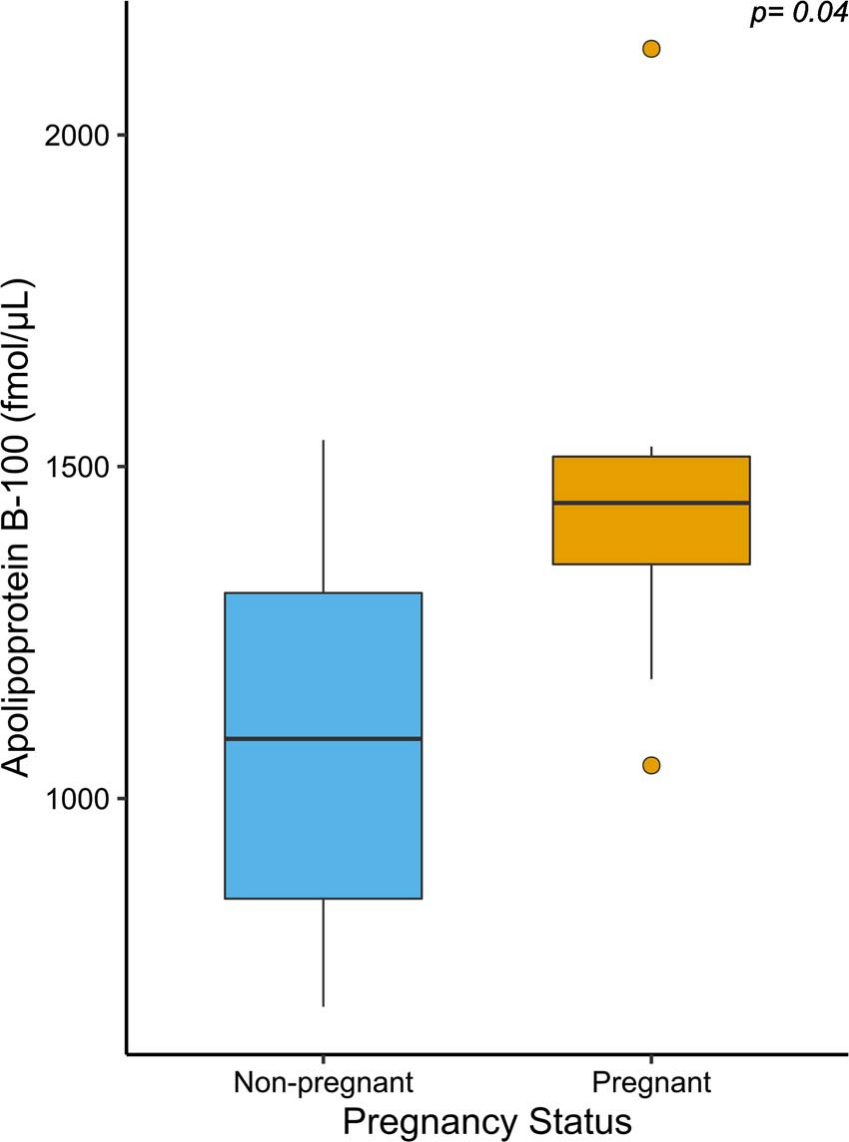
Serum expression of apolipoprotein B-100 in samples collected from female grizzly bears assumed to be non-pregnant (*n* = 9) and pregnant (*n* = 8) in the fall (August–October). The box-and-whisker plots show (i) the median represented by a thick horizontal line; (ii) the interquartile range represented by the box; (iii) the minimum and maximum values, excluding outliers, represented by the lower and upper whiskers; and (iv) outliers being less than or greater than 1.5 times the lower and upper quartiles, represented by the closed circles.

**Figure 3:**
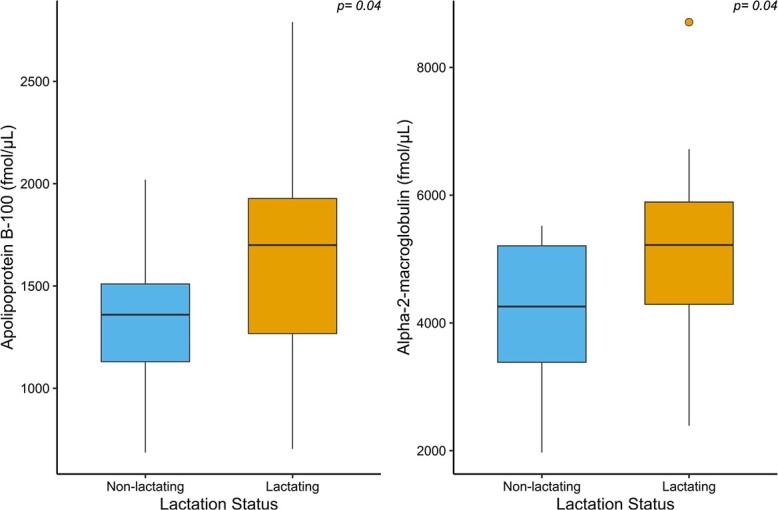
Serum expression of apolipoprotein B-100 and alpha-2-macroglobulin in female grizzly bears observed lactating (*n* = 22) and non-lactating (*n* = 14). The box-and-whisker plots show (i) the median represented by a thick horizontal line; (ii) the interquartile range represented by the box; (iii) the minimum and maximum values, excluding outliers, represented by the lower and upper whiskers; and (iv) outliers being less than or greater than 1.5 times the lower and upper quartiles, represented by the closed circles.

When considering all samples (*n* = 20 pregnant females; *n* = 20 non-pregnant females), we found that the median serum expression of afamin was significantly (*P* = 0.04) greater in samples collected from pregnant females than non-pregnant females (Figure 1). The median serum expression of alpha-2-macroglobulin, vitamin D binding protein and apolipoprotein B-100 were also marginally significantly (*P* < 0.1) elevated in samples collected from pregnant females compared to non-pregnant females. When analysing the subset of samples collected in the fall (August–October; *n* = 8 pregnant samples, *n* = 9 non-pregnant samples), the median serum expression of apolipoprotein B-100 was significantly (*P* = 0.04) greater in samples collected from pregnant females than non-pregnant females (Figure 2). The median expression of alpha-2-macroglobulin and apolipoprotein B-100 was significantly (*P* = 0.04) greater in samples collected from lactating females compared to non-lactating females (Figure 3).

**Figure 4:**
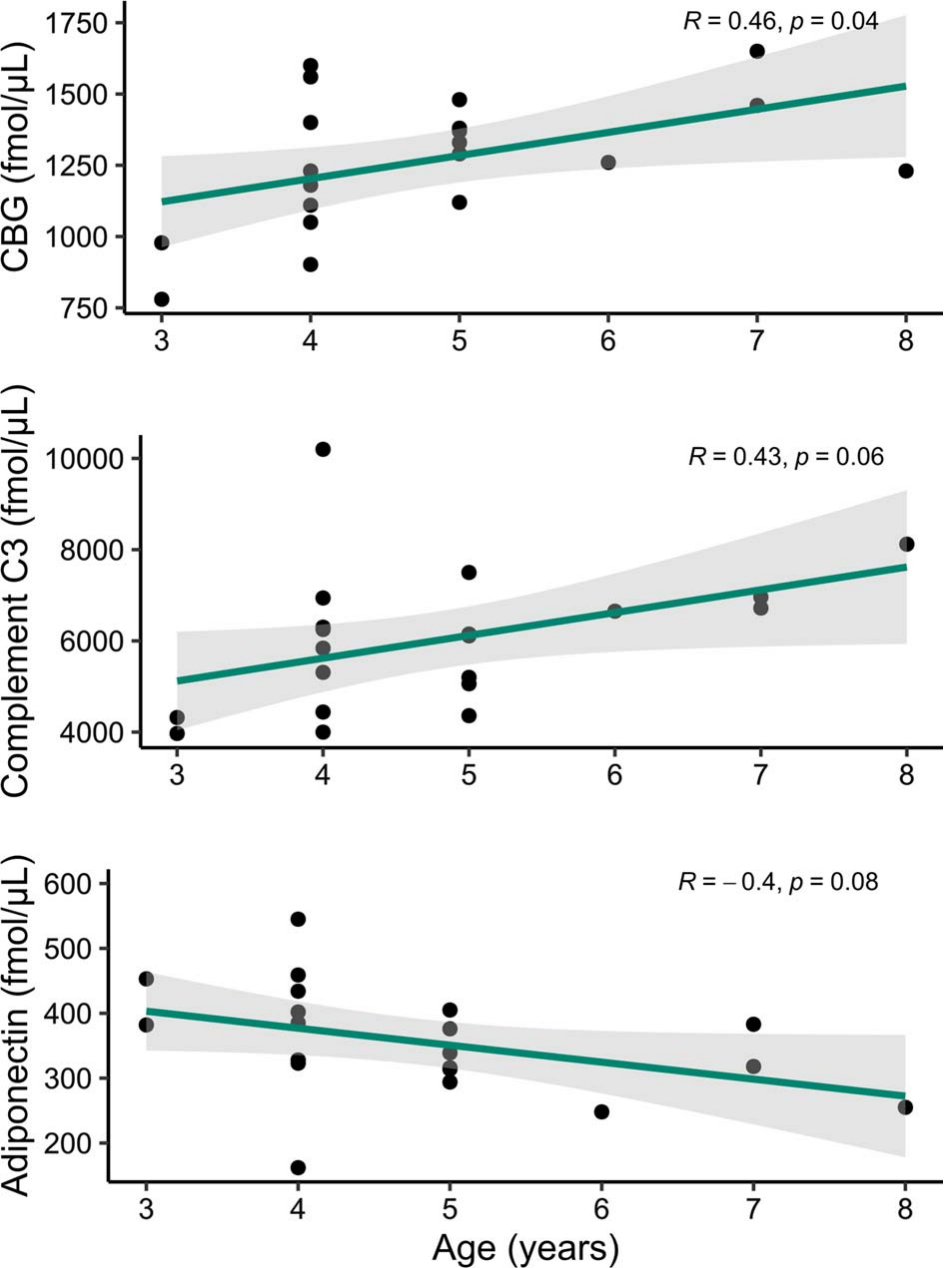
Serum expression of CBG, complement C3 and adiponectin in samples collected from subadult (<5 years) and adult (≥5 years) age classifications from the same female grizzly bear (*n* = 20 samples from 10 individuals). Grey shade is 95% confidence band around the Pearson’s linear relationship (line) and closed symbols are the raw data of protein expression.

**Figure 5:**
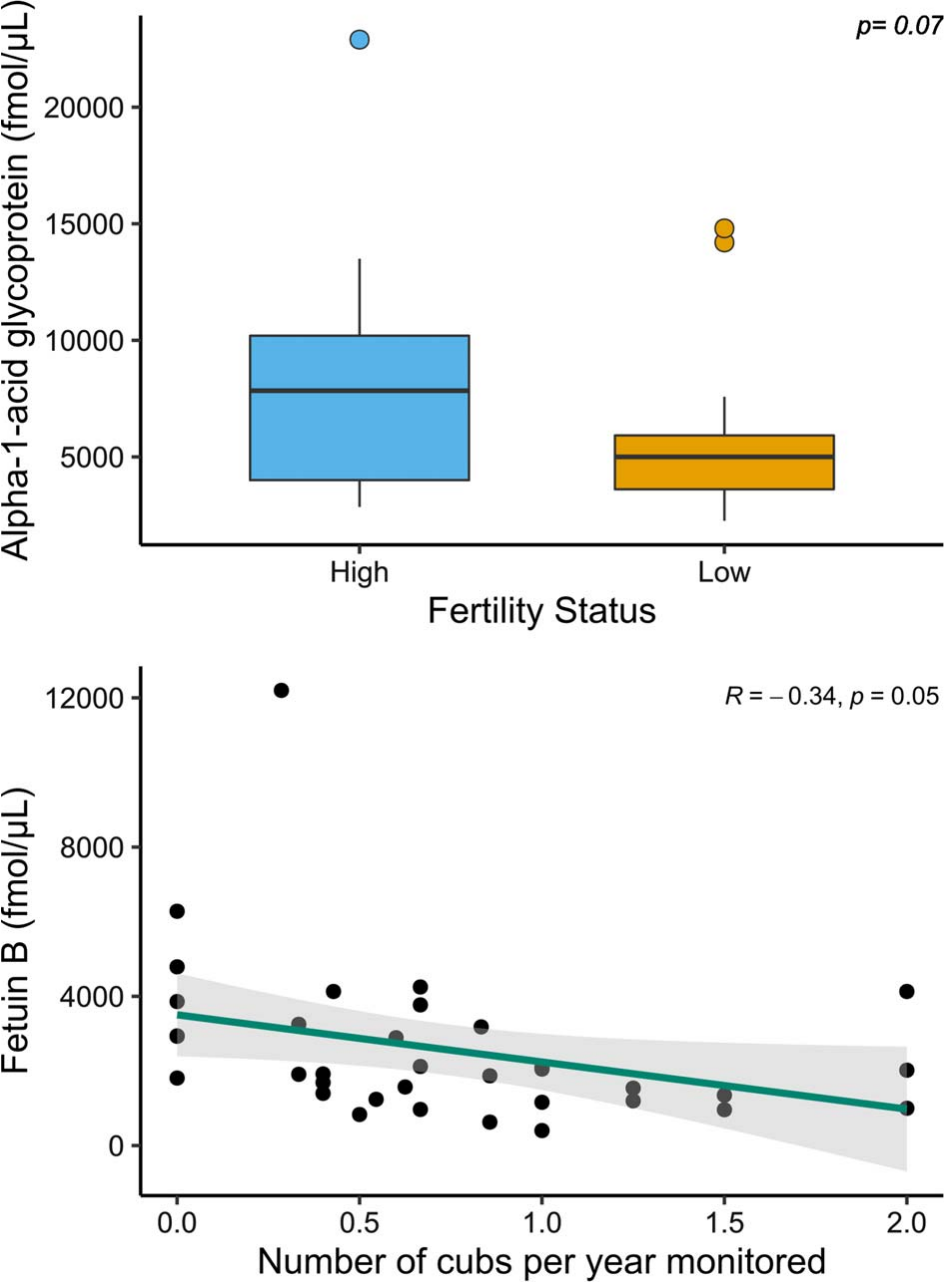
Serum expression of alpha-1-acid glycoprotein (top panel) and fetuin B (bottom panel) in samples collected from female grizzly bears classified as high (>0.73 cubs per year monitored; *n* = 14) reproducers compared to low (*n* = 21) and relative to the number of cubs per year monitored (*n* = 35). The box-and-whisker plots show (i) the median represented by a thick horizontal line; (ii) the interquartile range represented by the box; (iii) the minimum and maximum values, excluding outliers, represented by the lower and upper whiskers; and (iv) outliers being less than or greater than 1.5 times the lower and upper quartiles, represented by the closed circles. Grey shade is 95% confidence band around the Pearson’s linear relationship (line) and closed symbols are the raw data of protein expression.

**Figure 6:**
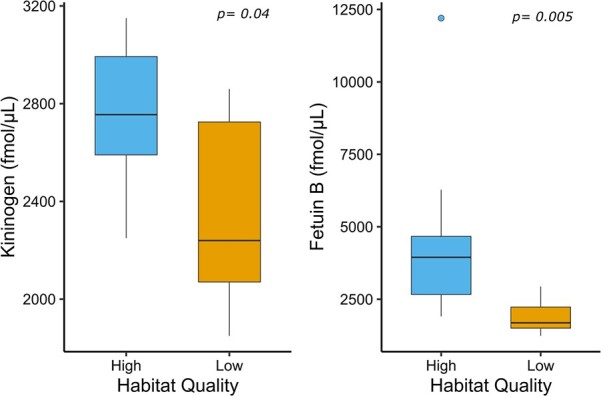
Serum expression of kininogen and fetuin-B was significantly (*P* < 0.05) greater in mothers and cubs that resided in high-quality (kilocalorie density, >70 000 000 kilocalorie per km2) habitat (*n* = 8 individuals) than those in low-quality habitat (*n* = 8 individuals). The box-and-whisker plots show (i) the median represented by a thick horizontal line; (ii) the interquartile range represented by the box; (iii) the minimum and maximum values, excluding outliers, represented by the lower and upper whiskers; and (iv) outliers being less than or greater than 1.5 times the lower and upper quartiles, represented by the closed circles.

**Figure 7:**
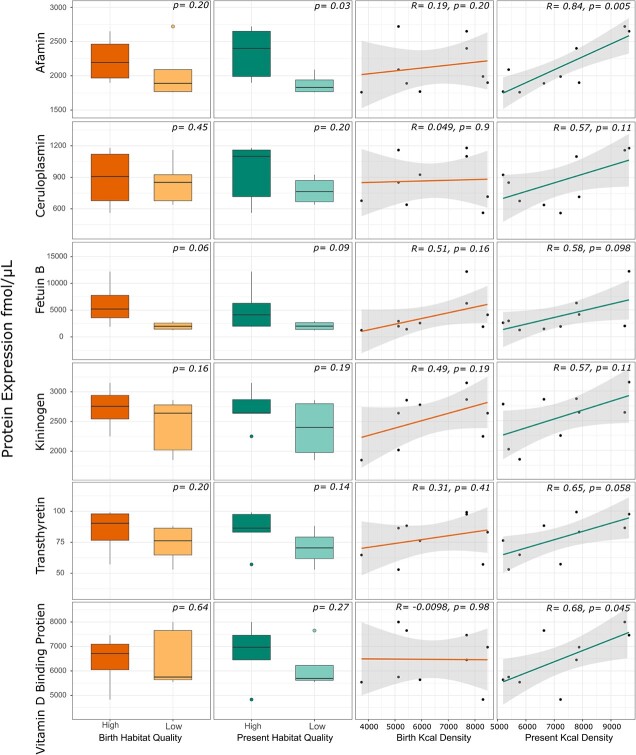
Serum expression of proteins related to energetics, reproduction and stress compared to habitat quality and kilocalorie (Kcal) density in cubs at birth (early life condition) and at present (time of sample collection). High-quality habitat was defined as kilocalorie density of >70 000 000 kilocalorie per km2. Only proteins with significant differences (*P* < 0.05) and correlations (*R* > 0.5) are shown. The box-and-whisker plots show (i) the median represented by a thick horizontal line; (ii) the interquartile range represented by the box; (iii) the minimum and maximum values, excluding outliers, represented by the lower and upper whiskers; and (iv) outliers being less than or greater than 1.5 times the lower and upper quartiles, represented by the closed circles. Grey shade is 95% confidence band around the Pearson’s linear relationship (line) and closed symbols are the raw data of protein expression.

### Sexual maturity

The median serum expression of all proteins did not significantly (*P* > 0.05) differ between samples collected at adult and subadult age categories from the same individual. The median serum expression of corticosteroid-binding globulin (CBG) was significantly (*R* = 0.46; *P* = 0.04) associated with increasing age (Figure 4). The median serum expression of adiponectin (*R* = −0.4; *P* = 0.08) and complement C3 (*R* = 0.43; *P* = 0.06) was marginally significantly correlated with increasing age (Figure 4).

### Fertility

The median serum expression of all proteins did not significantly (*P* > 0.05) differ between samples collected from high reproducers (>0.73 cubs per year monitored) and low reproducers. However, the median serum expression of alpha-1-acid glycoprotein was marginally significantly (*P* = 0.07) greater in samples collected from females classified as high reproducers than low reproducers (Figure 5). Additionally, the median serum expression of fetuin-B was marginally significantly (*R* = −0.34; *P* = 0.05) correlated with the number of cubs produced per years monitored (Figure 5).

### Silver spoon effects

We determined if protein expression was influenced by silver spoon effects by using 18 serum samples collected from 10 mother/daughter pairs. Corresponding mothers and cubs were both either below or above the mean Kcal density threshold for high- and low-quality habitat. No cubs that had a high-quality habitat during their early life then moved to a poor-quality habitat in their adult life and vice versa, meaning we could not test if fitness improved even if habitat quality worsened. When mothers and cubs were considered together, the median expression of kininogen and fetuin-B was significantly (*P* < 0.05) greater in bears that resided in high-quality (Kcal density, >70 000 000 Kcal/km^2^) habitat than those in low-quality habitat (Figure 6). Serum expression of proteins related to energetics, reproduction and stress relative to habitat quality (high vs. low) and Kcal density in cubs at birth (early life condition) and at present (time of sample collection) are shown in Figure 7. Serum expression of fetuin-B was marginally significantly (*P* = 0.06) greater during adulthood in cubs that spent their early life in high-habitat quality areas compared to low-habitat quality areas and was positively correlated with Kcal density in their early life habitat (*R* = 0.51); however, this correlation was not significant (*P* = 0.16). When considering the habitat quality of cubs where they presently reside, serum expression of afamin was significantly (*P* = 0.03) elevated in cubs in high-quality habitat than low-quality habitat and was positively correlated (*R* = 0.84; *P* = 0.005) with Kcal density in their present habitat. Serum expression of vitamin D-binding protein was also positively correlated with Kcal density in the present habitat of the individual (*R* = 0.68; *P* = 0.045). While not statistically significant (*P* > 0.05), median serum expression of transthyretin (*R* = 0.65), kininogen (*R* = 0.57), fetuin-B (*R* = 0.58) and ceruloplasmin (*R* = 0.57) was positively correlated with Kcal density in the present habitat of the individual.

## Discussion

Monitoring the productivity and reproductive characteristics of free-ranging wildlife is known to provide valuable information about the persistence of populations; however, methods are often lacking to do so within management programs. In this study, our goal was to monitor the productivity and reproductive characteristics of brown bears by identifying potential biomarkers of reproduction. If successful, these biomarkers would be useful in answering specific management-focused questions related to the reproductive performance of a population, including pregnancy, lactation status, sexual maturity and fertility. We found biomarkers of pregnancy (apolipoprotein B-100 and afamin), lactation (apolipoprotein B-100 and alpha-2-macroglobulin) and sexual maturity (CBG), but there were no statistically significant relationships with protein expression and fertility. There was also no evidence of silver spoon effects when using protein expression as a measure of fitness. This study highlights potential biomarkers of reproductive status and provides additional methods for monitoring physiological function and reproductive performance in wildlife.

Given that serum protein concentrations reflect a point in time and the current physiological state of the individual (Ray *et al.,* 2011), we expected the expression of proteins indicative of pregnancy that were measured in samples collected in the fall to increase around the time of implantation (November or December). Previous studies have shown that the mating season for brown bears in our study area extends from mid-May to the end of July and peaks in mid-June (Stenhouse *et al*., 2005); therefore, samples collected in early spring may have been from females before mating occurred. However, the ability to predict pregnancy prior to entering the den in November would be of great value for understanding individual reproductive performance and the number of cubs on the landscape the following year. Previous studies have reported increases in progesterone levels and body temperature in mated (assumed to be pregnant) females in December, which coincides with the timing of implantation (Dehnhard *et al.,* 2006; Friebe *et al.,* 2014). The expression of serum proteins related to energetics and reproduction, apolipoprotein B-100 and afamin was greater in samples collected from pregnant females than non-pregnant females. Apolipoprotein B-100 is the primary protein in low-density lipoprotein cholesterol, and therefore involved in metabolic function by transporting lipids from the digestive tract to various parts of the body (Bali and Utaal, 2019) and was differentially expressed when considering samples that were collected in the fall season (August–October). Afamin is a glycoprotein that is primarily sourced in the liver and is responsible for transporting vitamin E in body fluids such as plasma, ovarian follicular fluid, and seminal fluids, suggesting it plays a role in reproduction and more specifically, female fertility (Voegele *et al.,* 2002; Dieplinger and Dieplinger, 2015). Research in humans has shown that afamin concentrations increase during uncomplicated pregnancies (Hubalek *et al.,* 2014); however, increased afamin concentrations have also been shown to be an early predictor of pregnancy complications including preeclampsia and gestational diabetes mellitus in pregnant women (Hubalek *et al.,* 2014; Köninger *et al.,* 2018; Tramontana *et al.,* 2018). In humans, insulin sensitivity increases during early gestation to facilitate the uptake of glucose into adipose stores (Di Cianni *et al.,* 2003); however, later in gestation, the secretion of local and placental hormones promote insulin resistance, resulting in high glucose concentrations that are used to aid in the growth of the foetus (Catalano *et al.,* 1991). A similar pattern of insulin sensitivity and resistance has been documented in hibernating bear species throughout the year. During hibernation, non-pregnant captive bears were found to be insulin resistant, but had normal levels of glucose and were insulin sensitive during the spring and fall active periods when glucose storage and fat accumulation were high (Kamine *et al.,* 2011; McCain *et al.,* 2013; Rigano *et al.,* 2017). This insulin sensitivity and resistance pattern changes in different ways during both hibernation (when female bears are assumed to be pregnant) and pregnancy in mammals. Elevated afamin concentrations in samples collected from pregnant brown bears throughout the year (May–October) would suggest an increase in glucose storage due to increased insulin sensitivity; yet, afamin concentrations in humans have been shown to be elevated due to insulin resistance. Differences in the expression of compounds that regulate metabolic processes between humans and hibernating species have been previously identified, which may occur because of seasonal changes due to hibernation, delayed implantation and/or different physiologic processes (Rivet *et al.,* 2017).

In this study, we found that serum collected from lactating females showed an increase in the expression of apolipoprotein B-100. Given the role that apolipoprotein B-100 plays in cholesterol transport (mentioned above), it may serve as a promising biomarker for increased metabolic demand and lactation. During the denning period, female bears that gave birth utilize their lipid reserves accumulated in the fall to support metabolism and lactation for the following 1.5–2.5 years (Farley and Robbins, 1995; Oftedal, 2000; Steyaert *et al.,* 2012). This energy is provided by the transfer of lipids from adipose tissue in varying forms, including lipoproteins such as apolipoprotein B-100. Our findings are consistent with previous studies that found an increase in plasma lipid metabolites in lactating black bears (LeBlanc *et al.,* 2001) as well as apolipoproteins during lactation in cows (Kurpińska *et al.,* 2015). Furthermore, plasma cholesterol approximately doubled in bears that were denning compared to those that were active (LeBlanc *et al.,* 2001). We further found that the expression of alpha-2-macroglobulin was higher in serum collected from lactating females than non-lactating females. Alpha-2-macroglobulin is a large glycoprotein that plays a crucial role in the stress and immune response by binding to and regulating the activity of several different biologically important compounds, including zinc (Rehman *et al.,* 2013). Zinc is an essential trace element that is necessary for growth, development and immune function and is transferred to developing young during lactation (Kloubert and Rink, 2015; Djurović *et al.,* 2017). Given that zinc must be bound to alpha-2-macroglobulin in order for to circulate throughout the body, an increase in the expression of this protein during lactation may facilitate the transport of biologically important compounds in milk through lactation. A lactating female may be an indication of a successful birth and lactating females may still be accompanied by cubs, which provides information to managers regarding reproductive status, the presence of cubs on the landscape and population demographics. Therefore, using this methodology to determine lactation status may improve population monitoring techniques.

In our study animals, we found as age and sexual maturity increased from sub adult to adult, so did the expression of CBG. CBG has been used as a biomarker of stress in several species, including brown bears (Chow *et al.,* 2010; Wilson *et al.,* 2020b), as it is the protein primarily responsible for binding and transporting cortisol, a key hormone in the mammalian stress response (Meyer *et al.,* 2016). CBG can also regulate the availability of steroid hormones to tissues and direct the delivery of hormones to specific sites, as it has the ability to bind to progestins, androgens, oestrogens and mineralocorticoids (Breuner and Orchinik, 2002). While studies are limited on CBG and age relationships, previous data have shown differences in serum CBG expression in mammalian species, with higher levels typically found in mature females compared to males (Mataradze *et al.,* 1992; Fernandez-Real *et al.,* 2002; Chow *et al.,* 2010). Sex steroids (androgens and oestrogens) are thought to influence CBG concentrations in mature animals (Boonstra, 2005; White *et al.,* 2006), with increased oestrogen treatments leading to increased plasma concentrations of CBG in humans and guinea pigs (Spangler *et al.,* 1969). Taken together, the increase in stress as bears mature from dependent sub-adults to independent adults needing to search for suitable habitat, food and mates and the increase in sex hormones associated with sexual maturity may explain the observed increase in CBG found in the serum of samples collected from females before and after assumed sexual maturity.

The silver spoon hypothesis is a measure of how early life environments can affect individual development and states that individuals developing under favourable conditions (i.e. higher food resources) will have greater fitness benefits (Wilkin and Sheldon, 2009). Body size patterns of brown bears in the current study population have been shown to be influenced by habitat quality the year before and of the individual’s birth, suggesting the presence of silver spoon effects (Nielsen *et al.,* 2013). From a management perspective, habitat quality conditions in early life may forecast individual performance and ultimately population dynamics. However, the expression of proteins was more associated with the nutritional quality of their present habitat compared to their early life habitat, suggesting that protein expression was not useful in determining silver spoon effects for this population. This may be because protein concentrations in serum represent a current point in time and are thought to reflect the current physiological state of the individual, which can be instantly influenced by rapidly changing conditions, rather than change slowly over time (Ray *et al.,* 2011). Nevertheless, when considering present habitat, serum expression of afamin was elevated in cubs residing in habitat classified as high quality and this protein along with vitamin D-binding protein was positively correlated with Kcal density in their present habitat. Additionally, the median expression of kininogen and fetuin-B was significantly greater in all bears that resided in high-quality habitat than those in low-quality habitat. Both afamin and fetuin-B have been implicated as biomarkers of female fertility in previous studies (Voegele *et al.,* 2002; Dietzel *et al.,* 2016; Floehr *et al.,* 2016). Increased food resources in high-quality habitat has been shown to increase reproduction in other mammalian species (Chevallier *et al.,* 2020); therefore, the increase in the expression of proteins related to female fertility in the current study likely increased relative to the increase in nutritional quality of the landscape. This may also explain the inability to detect a potential biomarker for female fertility, as fertility is often dependent on food availability that can be variable across seasons and years. Vitamin D-binding protein is responsible for binding and transporting vitamin D metabolites, thus regulating the amount of vitamin D available in the body, which can influence growth and metabolism through the regulation of calcium and phosphorus (White and Cooke, 2000; Cline, 2012). Bears in our study area (interior bear populations) consume a mixed diet of green vegetation, berries and seasonal meat (Munro *et al.,* 2006), with few opportunities to ingest vitamin D rich sources, such as prey species including fish and ungulates (Cline, 2012). Ungulates provide a much higher concentration of energy for bears compared to vegetation, but they are often less abundant than vegetation and berries on the landscape (Coogan *et al.,* 2014). Furthermore, the expression of vitamin D-binding protein in the skin of the same brown bear population varied by season, suggesting differences in food availability (Wilson *et al.,* 2020b). An increase in the expression of vitamin D-binding protein in individuals residing in high-quality habitat may be indicative of richer food resources, such as ungulates, present in the area, as the ungulate Kcal content has been reported to vary across our study area (McClelland, *et al.* 2021). Differences in food availability across the landscape may also explain the increase in kininogen, a stress-related protein, relative to habitat quality, as food availability and stress have been linked to anthropogenic disturbance in this species (Bourbonnais *et al.*, 2013; Wilson *et al.,* 2020a, 2020b, 2021).

## Limitations

This study utilized a unique and robust long-term database of reproductive history from a sample of 49 female brown bears residing in five BMAs across Alberta, Canada. Based on the most recent population estimates for these areas, our study sampled 10% or more of adult females in each BMA (Alberta Environment and Parks, 2021). Depending on the population size, biologists may only need to collect samples from a few bears for monitoring purposes. Nevertheless, some reproductive characteristics are difficult to monitor over many years. For example, a female was assumed to be not pregnant if she was observed lactating at the time of sampling, with cubs at the time of sampling, or if she was observed with no cubs one year after sampling. However, females could have become pregnant and reabsorbed the fertilized embryo before implantation during hibernation or cubs could have been produced and lost prior to observation the following year. While the incidence of pregnancy loss, neonatal loss and first year cub mortality is unknown in this population, previous studies have reported sexually selected infanticide after den emergence as a source of cub mortality in other brown bear populations (reviewed by Steyaert *et al.,* 2012). Spontaneous lactation has been shown to occur in pseudopregnant females of mammalian species (Creel *et al.,* 1991; Gobello *et al.,* 2001; Zs *et al.,* 2010), making it difficult to distinguish a lactation cycle supporting cubs compared to one associated with a pseudopregnancy. In the current study, no cubs were observed in the surrounding area when six lactating females were captured; however, three were confirmed to have cubs based on our genetic database, tracking via ground or helicopter the previous year or trail camera images. Thus, we were unable to determine if a female was lactating as a result of producing cubs, a pseudopregnancy or a failed pregnancy. To investigate female fertility, we used the number of cubs produced during the years monitored as individuals were not monitored over their entire lifetimes, thus additional offspring could have been missed. Furthermore, we aimed to identify a biomarker that would indicate sexual maturity (the ability to reproduce); however, we did not have information on the age of actual first reproduction for those individuals. Additional factors known to influence resource availability and subsequently reproduction in this species, such as population density, anthropogenic disturbance and climate were also not taken into account. Our previous study found that the expression of several proteins identified as potential biomarkers in the current study was influenced by the year the sample was collected, BMA and season (Wilson *et al.*, 2020b); these factors were not considered in our statistical analysis due to limited sample size. Finally, several results were trending towards significant differential expression, suggesting the need for additional samples to detect statistically significant differences between groups. While this study provides an initial investigation and realistic methodology for monitoring the reproductive status of free-ranging brown bears using protein biomarkers, we suggest additional research is needed using closely monitored and/or captive individuals to be able to collect serial serum, hair and skin samples from individuals with known reproductive status and characteristics. In the future, we also suggest using similar methodology to identify reproductive biomarkers in samples that can be collected non-invasively (e.g. hair and skin), so that the potential negative effects of capture and handling on health can be avoided.

## Conclusions

This study identified potential protein biomarkers of reproductive status in serum collected from free-ranging female brown bears and used these proteins to address specific management-focused topics related to pregnancy, lactation and sexual maturity. We suggest that this information may be used to expand the conservation physiology toolbox by better understanding population demography, identifying reproductive status and forecasting the ability of individuals to reproduce. From a management standpoint, protein biomarkers can be used as a conservation tool to acquire additional information beyond observing cubs on the landscape to understanding the current and future reproductive potential within the population. Further research and validation may lead to developing tools to know the percentage of adult females within a population that are able to reproduce, the number of females that become pregnant in a year and/or the percentage of females that give birth (assumed from detection of recent lactation), but lose their cubs. Applications of this technique in the future should also consider non-invasive sampling methods (e.g. hair and/or skin). Ultimately, using and understanding the expression of protein biomarkers related to reproduction will help to develop conservation and management strategies for vulnerable populations and allow us to predict the likelihood that populations will persist long into the future.

## Funding

This work was supported by the Grizzly-PAW project (Natural Sciences and Engineering Research Council of Canada [File: CRDPJ 486175-15], Grantee: N.C. Coops, FRM, UBC), in collaboration with fRI Research and FRIAA, Alberta Newsprint Company, Canfor, Cenovus, Repsol, Seven Generations Energy, Shell Canada, TransCanada Pipelines, Teck Resources, West Fraser, Westmoreland Coal and Weyerhaeuser. More information can be found at http://paw.forestry.ubc.ca/.
